# Sub‐Diffraction Nanolithography of Halide Perovskite via Reversible All‐Optical Crystallization–Decomposition

**DOI:** 10.1002/adma.73939

**Published:** 2026-07-04

**Authors:** Zhengfen Wan, Xiao Huang, Fangyi Zhang, Qiming Zhang, Min Gu

**Affiliations:** ^1^ School of Artificial Intelligence Science and Technology University of Shanghai for Science and Technology Shanghai China; ^2^ Institute of Photonic Chips University of Shanghai for Science and Technology Shanghai China; ^3^ School of Electrical Engineering Computing and Mathematical Sciences Curtin University Perth Western Australia Australia

**Keywords:** halide perovskite, laser direct writing, photodetector, reversible modulation, sub‐diffraction nanolithography

## Abstract

Halide perovskites exhibit exceptional optoelectronic properties, yet their intrinsic chemical fragility and ionic nature pose fundamental challenges for high‐resolution patterning and nanoscale integration. Here we report a reversible, all‐optical structural modulation strategy that enables chemistry‐free, sub‐diffraction patterning of halide perovskite thin films through light‐driven crystallization‐decomposition dynamics. Localized femtosecond laser excitation induces controlled crystallization and an orthorhombic‐to‐cubic phase transition in CsPbBr_3_ thin films, markedly enhancing crystallinity and optoelectronic response. In contrast, ultraviolet illumination promotes defect formation and partial decomposition into CsBr and PbBr_2_, reversibly suppressing crystallinity and photocurrent. This optically driven crystallization‐decomposition cycle is repeatable over multiple iterations with more than 85% photocurrent recovery, establishing a robust platform for reversible material‐state control. Leveraging the nonlinear competition between laser‐induced crystallization and UV‐induced inhibition, we further demonstrate resist‐free, sub‐diffraction nanolithography with feature sizes down to 93.5 nm, well beyond the conventional optical diffraction limit. This work reveals a light‐programmable structural degree of freedom in halide perovskites and provides a general materials framework for reconfigurable perovskite architectures, adaptive photonics, and dynamic optoelectronic systems.

## Graphical abstract

A reversible, all‐optical modulation strategy enables resist‐free, sub‐diffraction nanolithography of halide perovskite films. Leveraging 800 nm fs laser‐induced crystallization and 365 nm laser driven decomposition, reconfigurable patterning achieves feature sizes down to 93.5 nm. This chemistry‐free approach offers dynamic control over optoelectronic states, paving the way for adaptive nanophotonics and programmable optoelectronic devices.

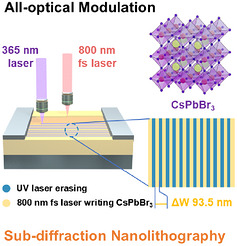

## 1 Introduction

Halide perovskites have emerged as highly versatile materials distinguished by exceptional light‐matter interactions [1], diverse crystal structures, and tunable electronic properties [2], leading to remarkable advances in photovoltaics, light‐emitting diodes (LEDs), lasers, and photodetectors [3, 4, 5]. As device dimensions approach the nanoscale, however, further advances increasingly depend on the ability to pattern perovskite thin films with high resolution, high structural fidelity, and minimal material degradation [6]. Nanoscale patterning is essential not only for device miniaturization, but also for accessing new physical regimes in light‐matter interaction, carrier transport and photonic confinement. However, realizing high‐resolution and scalable nanolithography in perovskites remains a fundamental challenge.

Considerable efforts have been devoted to patterning perovskite thin films, including photolithography [7, 8, 9], electron‐beam lithography [10], inkjet printing [11], and nanoimprint lithography [12]. While these approaches have enabled microscale or even nanoscale patterning, they are fundamentally constrained by the chemical and mechanical fragility of perovskites. Exposure to photoresists, solvents, moisture, and mechanical pressure often induces ion migration, interfacial damage, defects, and irreversible phase degradation [13], resulting in compromised optoelectronic performance and poor pattern reliability. Consequently, reliable nanoscale and particularly sub‐diffraction lithography of perovskite thin films remains inaccessible, severely limiting the translation of perovskites into nanoscale device platforms.

Laser direct writing (LDW) perovskite has recently emerged as a promising approach [14, 15], offering spatial selectivity, high flexibility, and compatibility with large‐scale fabrication [16]. Through localized photothermal effects, laser irradiation can trigger chemical reactions among perovskite precursors, enabling in situ crystallization and patterning of perovskite [17, 18, 19]. Importantly, three‐dimensional(3D) femtosecond(fs) LDW of CsPb(Br_1‐x_I_x_)_3_ within glass matrices enables nanoscale control over halide composition and bandgap engineering [20, 21]. Nevertheless, such approaches typically rely on electrically insulating hosts, which restrict charge transport and limit broader optoelectronic applications. Moreover, laser‐induced processes have predominantly been exploited in a unidirectional manner, focusing on permanent crystallization or modification rather than reversible modulation.

Notably, the same light‐matter interaction that drives crystallization can also trigger photo‐decomposition in halide perovskites, owing to their intrinsically low formation energies [22, 23]. Prior studies have demonstrated that CsPb(Cl/Br)_3_ nanocrystals embedded in glass can be decomposed by laser irradiation [24], highlighting the dual role of optical excitation in both formation and degradation. This duality suggests an opportunity for reversible optical modulation and nanolithography of perovskite.

Here, we report a fully optical and reversible nanolithography platform for CsPbBr_3_ perovskite thin films based on the complementary use of 800 nm fs laser‐induced crystallization (LIC) and ultraviolet (UV) light‐induced decomposition. The 800 nm fs laser induced crystallization of CsPbBr_3_ thin films and a phase transition from the orthorhombic to cubic structure results in a two orders of magnitude enhancement in photocurrent. Conversely, 365 nm UV exposure generates defects and decomposes CsPbBr_3_ into CsBr and PbBr_2_, effectively erasing the laser‐written patterns and suppressing the photocurrent. This crystallization‐decomposition process is repeatable for at least five cycles with over 85% recovery, demonstrating excellent reversibility and stability. Leveraging this dual‐light modulation, we achieve rewritable, sub‐diffraction lithography with feature sizes down to 93.5 nm with a dual‐beam laser system, well below the optical diffraction limit, while maintaining electrical functionality.

This reversible crystallization‐decomposition framework establishes a new paradigm for resist‐free, reconfigurable nanolithography in halide perovskites. Beyond enabling sub‐diffraction patterning, this optically programmable platform enables rewritable optoelectronic encoding and provides mechanistic insight into photo‐driven structural dynamics of perovskite at the nanoscale. It provides a route toward dynamically programmable optoelectronic architectures, with implications for nanoscale photonics, high‐density data storage, and adaptive device systems.

## 2 Results and Discussion

**FIGURE 1:**
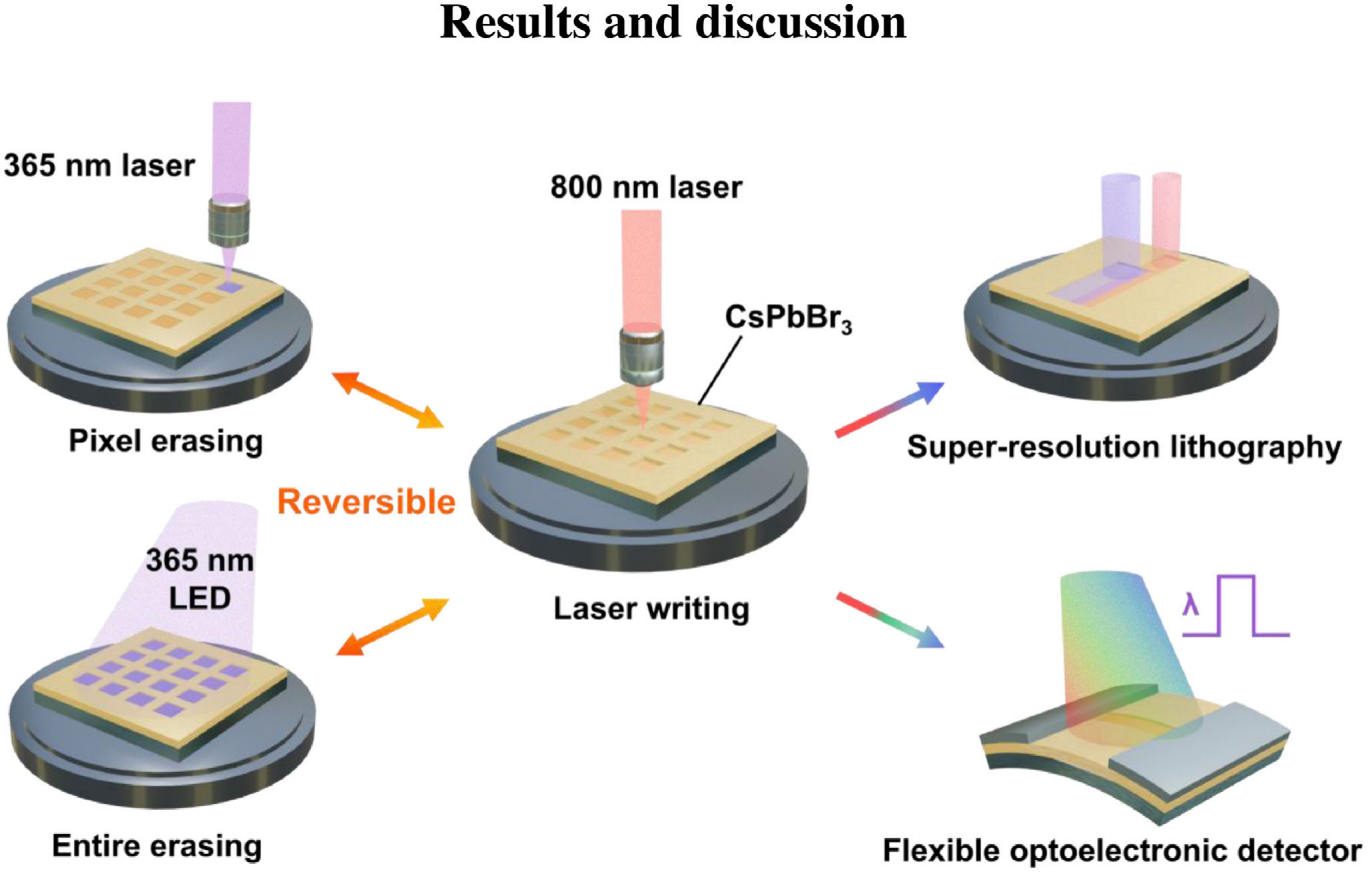
Schematic diagram of the laser writing and UV erasing CsPbBr_3_ thin films for photodetecting and super‐resolution lithography.

Figure 1 presents a schematic of the reversible optical modulation of CsPbBr_3_ thin films for photodetector applications and dual‐beam lithography. The CsPbBr_3_ films were prepared on glass substrates via a one‐step spin‐coating method using the perovskite precursor solution. Two distinct light sources were employed to tailor the film properties reversibly. An 800 nm fs laser‐induced crystallization within the CsPbBr_3_ layer, enhancing its optoelectronic performance. Conversely, exposure to 365 nm UV light degraded the perovskite, leading to diminished optoelectronic responses. Under controlled conditions, these two optical processes are fully reversible, enabling precise “writing” and “erasing” of the film's photoresponse for data encryption. By combining the 800 nm fs laser for writing and the 365 nm UV laser for erasing, the linewidth of the LIC can be tuned by adjusting the overlap of the two beams. The optical path of the 800 nm femtosecond laser system is illustrated in Figure S1.

**FIGURE 2:**
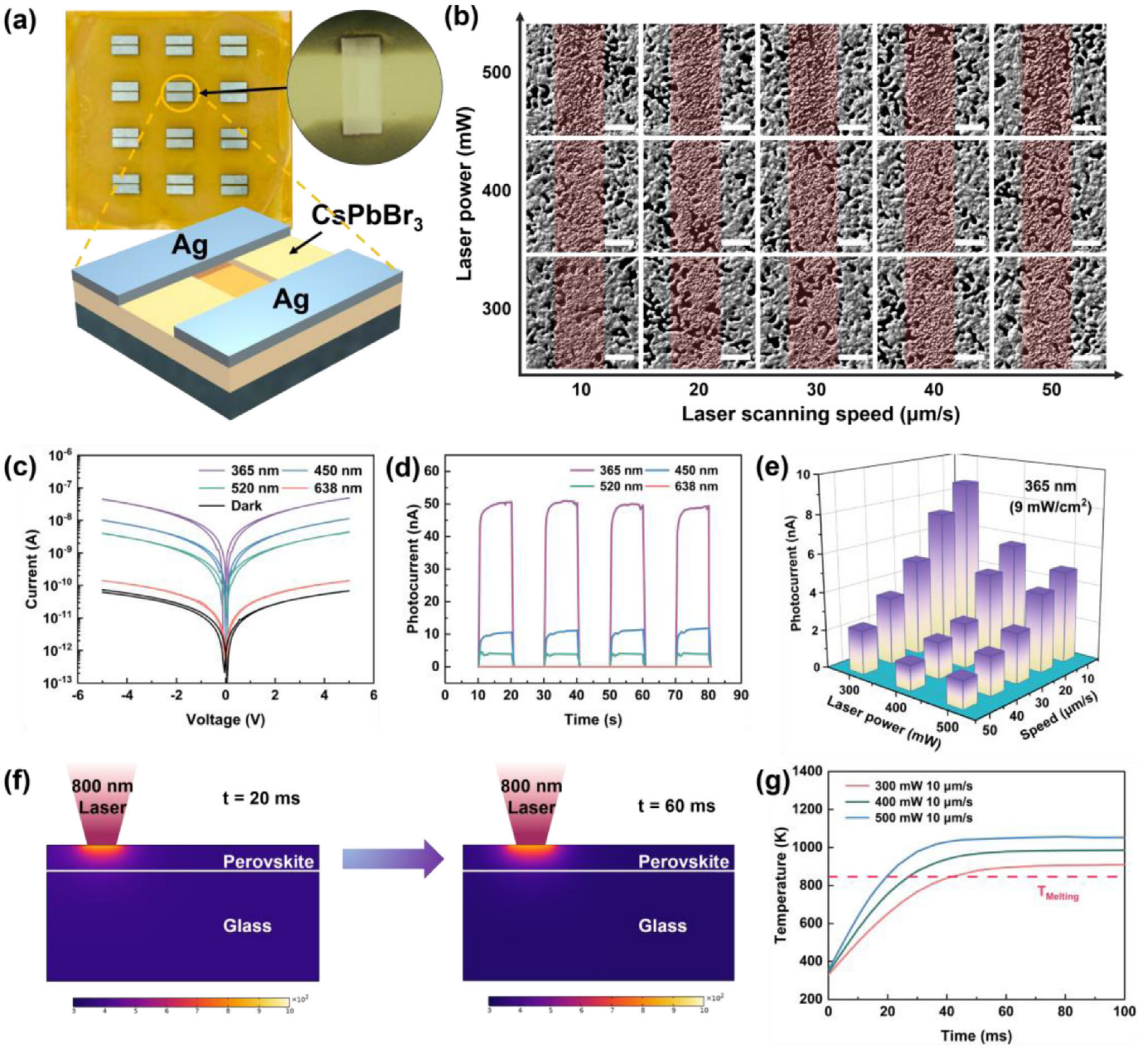
(a) Images and structural diagram of CsPbBr_3_ perovskite photodetectors, with the inset showing a microscopic image of the LIC. (b) SEM images of CsPbBr_3_ films processed under an 800 nm fs laser with different powers and scanning speeds, and the laser‐processed areas marked in red (scale bar: 1 µm). (c) The *I*–*V* curve of the LIC photodetector irradiated by different wavelengths of 365, 450, 520, and 638 nm at 200 mW/cm^2^. (d) The *I*–*T* curve of the device under illumination at different wavelengths of 365, 450, 520, and 638 nm at 200 mW/cm^2^. (e) Photocurrents of photodetectors fabricated with different laser processing parameters at wavelengths of 360 nm. (f) Temperature simulation of the sample irradiation by the 800 nm fs laser with 300 mW and 10 µm/s. (g) The simulated temperature of the sample irradiated by the 800 nm laser with powers of 300–500 mW.

Figure 2a presents a photograph of the CsPbBr_3_ photodetectors fabricated via 800 nm fs LDW, together with a schematic illustration of the device structure and a corresponding microscopic image (inset). The two‐terminal photodetector comprises a LIC region serving as the functional layer and two Ag electrodes. Figure 2b shows SEM images of CsPbBr_3_ films processed by an 800 nm fs laser under various laser powers and scanning speeds. Compared with the unprocessed CsPbBr_3_ (UC), the LIC regions exhibit well‐defined boundaries and a characteristic fish‐scale morphology, attributed to localized melting and recrystallization induced by the laser. The laser processing parameters significantly influence the perovskite morphology: laser power governs the delivered energy, where excessive power leads to ablation and degradation, while insufficient power fails to trigger effective recrystallization, resulting in limited defect healing. The LIC fabricated at 500 mW exhibits a granular texture, indicative of excessive degradation, whereas a laser power of 300 mW yields uniform microcrystals. In contrast, the laser scanning speed determines the heat accumulation and cooling rates during processing, which in turn affect the uniformity of recrystallization. The higher speeds result in rougher grain structures within the processed regions.

The optoelectronic properties of the LICs fabricated under different laser parameters were evaluated. Figure 2c,d indicate the I‐V and *I*–*T* curves of the LIC photodetectors under illumination at 365, 450, 520, and 638 nm. After laser processing, the LIC device exhibits approximately 100‐fold enhancement in photocurrent response at 365 nm UV light, with the photocurrent gradually decreasing as the incident wavelength increases. This behavior aligns with the absorption edge of CsPbBr_3_ (∼540 nm), resulting in a weak response in the red light region. Considering that high‐intensity 365 nm illumination can degrade the perovskite material, and Figure S2 has confirmed that an illumination intensity of 9 mW/cm^2^ does not adversely affect the perovskite within the test duration, all subsequent experiments were conducted using a 365 nm light source with a power density of 9 mW/cm^2^. To optimize the laser parameters, LIC photodetectors were fabricated at various laser powers and scanning speeds. As shown in Figure 2e, the device processed at 300 mW and 10 µm/s exhibits the highest photocurrent at 365 nm, a trend consistent across 450 nm, 520 nm, and 638 nm wavelengths (Figure S3a–c). Figure S4 further confirm that the processing condition of 300 mW and 10 µm/s is optimal. Therefore, 300 mW and 10 µm/s were adopted as optimal parameters for subsequent fabrication. Figures S5 and S6, respectively, show the *I*–*T* curves of the photodetector before and after processing with the optimal laser parameters. After laser processing, the LIC device displays an approximately 100‐fold enhancement in photocurrent response at 365 nm, while maintaining excellent pulse stability across wavelengths. In addition, three key parameters of LIC‐based photodetectors: responsivity (*R*), external quantum efficiency (EQE), and specific detectivity (*D*) were calculated, as shown in Figure S7a–c. Notably, under 365 nm illumination, the *R* and EQE of the device can reach 1.24 × 10^−2^ A/W and 4.22%, respectively. A comparison of the CsPbBr_3_ photodetectors prepared with different methods is demonstrated in the Table S1.

To investigate local thermal behavior during LDW, laser‐material interaction simulations were performed using COMSOL Multiphysics. The simulation procedure and underlying principles are detailed in the Supporting Information. When the laser beam with a Gaussian distribution hits the surface of the perovskite layer, a portion of the laser energy is absorbed and converted to an instantaneous local heating. This process can be described by the Fourier heat conduction law and the Beer–Lambert law [25, 26]:

(1) $$\rho C_{p}\frac{\partial T}{\partial t}+\rho C_{p}u\cdot \nabla T+\nabla \cdot q\;=Q$$

(2) q=−k∇T

(3) $$\frac{\partial I}{\partial y}=\alpha \left(T\right)I$$

The thermal annealing process and the temperature distribution within the CsPbBr_3_ film during laser processing were simulated. As shown in Figure 2f, the simulated temperature distribution reveals that at ∼60 ms, the laser‐induced peak temperature exceeds 900 K and remains stable thereafter. Figure 2g also presents the temperature evolution over time under different laser powers. It can be noticed that when the laser power reaches 300 mW, the peak temperature already exceeds the melting point of CsPbBr_3_ (T_melting = 847.15 K). As the laser power increases, both the heating rate and the peak temperature of the CsPbBr_3_ film increase accordingly. However, excessive laser power can lead to ablation of the perovskite material, resulting in degradation and mass loss. The simulations confirm that 300 mW is an optimal power for controlled laser annealing, consistent with experimental findings.

Compared with conventional thermal annealing, LDW provides several key advantages for the patterning and processing of perovskite thin films. It enables mask‐free, high‐resolution photopatterning with localized recrystallization and defect engineering at the micrometer scale. As shown in Figure S8a–c, absorption, photoluminescence (PL), and x‐ray diffraction (XRD) analyses reveal that laser‐processed films exhibit more substantial absorbance, reduced defect density, and enhanced crystallinity compared to those treated with thermal annealing. Photocurrent measurements (Figure S10) further demonstrate superior photoresponse in laser‐annealed samples across all wavelengths, highlighting the superior optoelectronic enhancement achieved by LDW. Furthermore, LDW is highly compatible with thermally sensitive substrates, such as polyethylene terephthalate (PET). Flexible CsPbBr_3_ photodetectors fabricated on PET substrates display stable and repeatable photoresponses (Figure S11). The devices maintain complete mechanical flexibility, withstanding bending angles up to 90°, and their photocurrent increases with the increase of the bending degree. After 300 bending cycles, responsivity and detectivity remain above 80%. Moreover, after 35 days of storage in argon conditions, the photocurrent retains over 95% of its initial value, demonstrating excellent mechanical and environmental stability.

**FIGURE 3:**
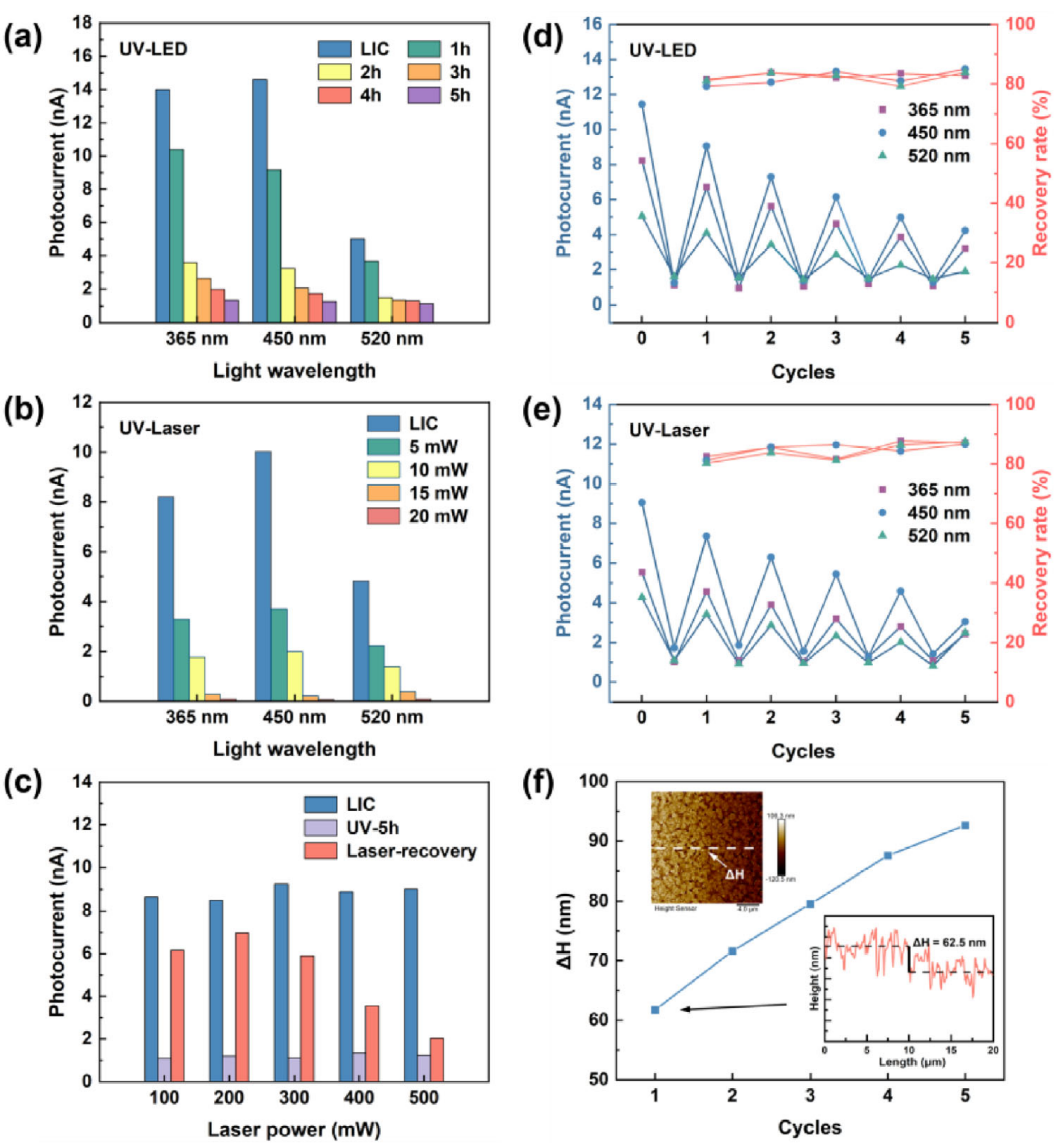
(a) Photocurrent variation of LIC photodetectors treated under different durations of UV LED light. (b) Photocurrent variation of LIC photodetectors treated with different UV laser powers. (c) Recovery process of UV‐LIC 5 h under different powers of the 800 nm fs laser. (d,e) Photocurrent and recovery rate of a LIC photodetector with the UV LED erasing process (d) and the UV laser erasing process (e) under different recovery cycles. The power intensity of illumination: 365 nm (9 mW/cm^2^), 450 nm (200 mW/cm^2^), and 520 nm (200 mW/cm^2^). (f) Height loss of the LIC region during the writing‐erasing‐recovery process. The inset shows an AFM image and the height changes of the thin film after the first writing‐erasing‐recovery cycle.

To erase the optoelectronic performance of the LIC films, 365 nm UV light was employed to irradiate the LIC photodetectors under ambient conditions. Figure 3a shows the photocurrent evolution of the LIC photodetectors after 1 to 5 h of 365 nm UV LED light exposure. A pronounced decline in photocurrent is observed, with the most significant decrease occurring within the first 1–2 h. With prolonged exposure, the photocurrent of the UV‐treated LIC (UV‐LIC) continues to decrease, ultimately dropping by approximately 90% after 5 h of treatment, effectively “erasing” the film's optoelectronic response. Similarly, a 365 nm UV laser was employed to achieve a controlled erasing process. As shown in Figure 3b, the extent of photocurrent suppression can be tuned by varying the UV laser power. Increasing the UV laser power from 5 to 20 mW results in a progressive decrease in photocurrent. After the erasing process, the 800 nm fs laser was applied to recover the optoelectronic response of the UV‐LIC devices (Figure 3c). Various laser parameters were applied, and a scanning speed of 10 µm/s and a laser power of 200 mW demonstrate optimized recovery effects.

To assess the reproducibility of the reversible modulation, repeated “writing‐erasing” cycles were performed on CsPbBr_3_ photodetectors, and the photocurrent evolution was monitored. Figure 3d shows the cyclic response under 800 nm fs laser writing and 365 nm UV LED erasing (5 h exposure), while Figure 3e presents the results obtained using UV laser erasing (10 mW, 10 µm/s). In both cases, the recovered photocurrent consistently retained over 85% of its previous value through five consecutive cycles under illumination at 365, 450, and 520 nm, confirming the robustness and reproducibility of the reversible process.

The thickness evolution of the perovskite film during the repeated modulation cycles was also characterized using atomic force microscopy (AFM), as shown in Figure 3f. The first “writing‐erasing‐recovery” cycle reduced the film thickness by approximately 62.5 nm. Subsequent “writing‐erasing” cycles caused an additional reduction of ∼8 nm per cycle. These results indicate that the process introduces incremental material loss, which can eventually impact the effectiveness of recovery over multiple cycles.

**FIGURE 4:**
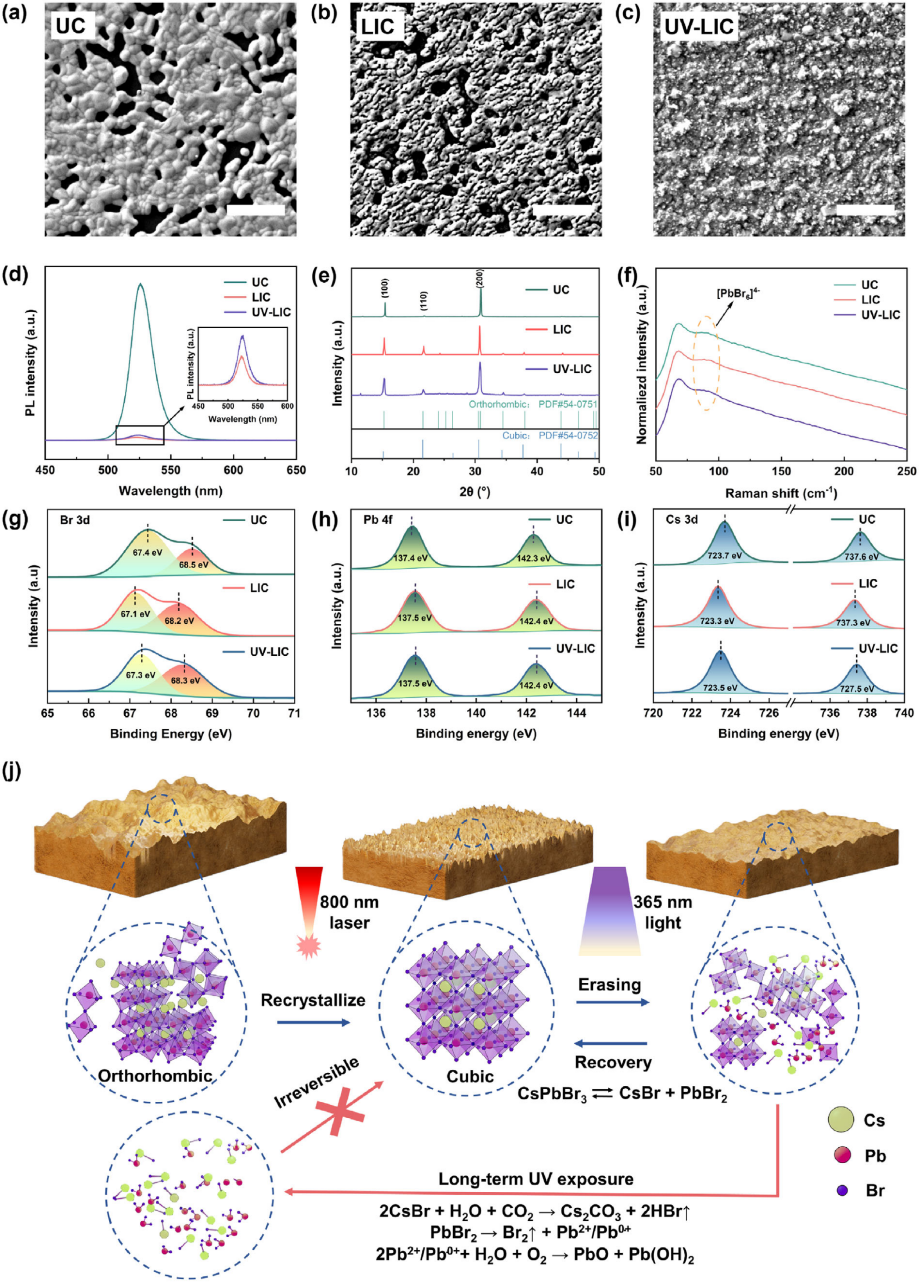
The SEM images of (a) UC, (b) LIC, and (c) UV‐LIC (scale bar:1 µm). (d) PL spectra of UC, LIC, and UV‐LIC. (e) XRD spectra of UC, LIC, and UV‐LIC. (f) Raman spectra of UC, LIC, and UV‐LIC. High‐resolution XPS spectra corresponding to (g) Br 3d, (h) Pb 4f, and (i) Cs 3d of CsPbBr_3_ thin films. (j) Schematic diagram of the effects of erasing‐recovery process on CsPbBr_3_ perovskite thin films.

To further elucidate the effects of 800 nm laser writing and 365 nm UV erasing on the optical and structural properties of the unprocessed CsPbBr_3_ (UC), LIC, and UV‐LIC were characterized (Figure 4a–i). Figure 4a–c shows SEM images of the UC, LIC, and UV‐LIC samples, respectively. It can be observed that UC exhibits distinct grains with obvious boundaries between crystals. After 800 nm fs laser processing, the morphology of the LIC film presents a dense fish‐scale pattern with obscure grain boundaries. The laser‐induced melting and recrystallization process significantly modifies the surface morphology of the thin film. After laser treatment, the LIC demonstrates an enhanced absorption coefficient, as shown in Figure S12. Laser melting causes adjacent grains to crosslink, forming larger grains and thereby enhancing the light absorption capability. Meanwhile, a large number of nanocrystals are generated on the film surface. These nanocrystals fill the grain boundary voids, resulting in narrower and fewer grain boundaries. The fish‐scale morphology further facilitates carrier transport by enabling photogenerated carriers to accumulate at the surface tips and undergo hopping transport once a critical density is reached [27], markedly improving carrier mobility and photocurrent response. In contrast, UV exposure decomposes CsPbBr_3_ into CsBr and PbBr_2_, disrupting the crystalline network. The migrated CsBr aggregates on the surface, inducing grain agglomeration and generating defects that impede carrier transport and promote non‐radiative recombination.

Figure 4d presents the PL spectra of UC, LIC, and UV‐LIC. After laser processing, the PL intensity of the film is significantly quenched. To explore the mechanism of the quenched PL, the space‐charge‐limited current (SCLC) and time‐resolved photoluminescence (TRPL) measurements were conducted. Figure S13 shows that after laser processing, defect density were reduced and carrier lifetime prolonged, suggesting that enhanced non‐radiative recombination is unlikely to dominate the PL quenching in the LIC film. Combined with the SEM morphology analysis, the laser‐induced melting and recrystallization process effectively reduces the number of grain boundaries between adjacent grains and promotes the formation of crosslinked structures among the grains. Therefore, the PL quenching in the LIC film can be primarily attributed to the enhanced exciton dissociation with laser‐induced structural evolution. In contrast, UV treatment significantly increases the defect density and shortens the PL lifetime, indicating that the suppressed PL intensity in the UV‐LIC film mainly originates from defect‐assisted non‐radiative recombination induced by photodegradation [28]. Additionally, the PL peak exhibits a blue shift from 526.1 to 522.6 nm, indicating a widening of the bandgap. This shift arises from the suppression of indirect recombination, as laser recrystallization passivates shallow‐level defects that typically act as non‐radiative centers.

Figure 4e shows the XRD patterns of UC, LIC, and UV‐LIC. The characteristic peaks at 15.24°, 21.5°, and 30.7° correspond to the (100), (110), and (200) planes of CsPbBr_3_, respectively. Notably, the (110) peak intensity increases significantly after laser processing, suggesting that laser‐induced recrystallization promotes preferential growth along the (110) plane. Since ion migration in perovskites primarily occurs along (110) directions, this orientation facilitates efficient charge transport and reduces trap states, contributing to enhanced photocurrent [29, 30]. Moreover, after laser processing, the full width at half maximum (FWHM) of the (200) peak decreases from 0.18° to 0.11°, indicating improved crystallinity and fewer defects [31]. A concurrent phase transition from orthorhombic to cubic symmetry is observed, which further enhances carrier mobility due to reduced lattice distortion and higher symmetry [32, 33]. Conversely, UV exposure decreases the (110) peak intensity and broadens the (200) peak, while additional peaks at 11.3° and 28.5° confirm the formation of PbBr_2_, a degradation byproduct. Raman spectra (Figure 4f) for all samples exhibit a characteristic peak at 75 cm^−1^, corresponding to the [PbBr_6_]^4+^ octahedra. The reduced intensity in UV‐LIC indicates partial perovskite decomposition, consistent with XRD observations.

To further investigate the chemical components in CsPbBr_3_ films during the laser writing and UV erasing processes, x‐ray photoelectron spectroscopy (XPS) of the UC, LIC, and UV‐LIC samples was analyzed. The XPS survey spectra confirm the presence of key elements C, Cs, Pb, and Br in all three samples, as shown in Figure S14. High‐resolution XPS spectra of Br 3d, Pb 4f, and Cs 3d for the three samples are presented in Figure 4g–i. For UC, the binding energies of Br 3d_5/2_, Pb 4f_7/2,_ and Cs 3d_5/2_ are located at 67.4, 137.5, and 723.7 eV, respectively, which are consistent with previous studies [34]. After laser treatment, the Br 3d and Cs 3d peaks shift to lower binding energies, while the Pb 4f peak exhibits only a negligible shift. In CsPbBr_3_, Pb^2+^ is located at the center of the [PbBr_6_]^4−^ octahedron, and its coordination number and bond lengths are relatively stable [35, 36]. Therefore, even when a phase transition from orthorhombic to cubic occurs, the change in the binding energy of Pb is very limited. In contrast, we attribute the shifts of the Br 3d and Cs 3d peaks mainly to the shortening of the Cs─Br bond length and the increase in electron cloud density when the lattice transforms from orthorhombic to cubic [37, 38, 39]. After 5 h of UV irradiation, the Br 3d and Cs 3d peaks shift to higher binding energies by approximately 0.3 eV, while the Pb 4f peak shows only a very small shift (< 0.1 eV). Since Pb exists as Pb^2+^ in both CsPbBr_3_ and PbBr_2_, the difference in their binding energies is small; thus, before further decomposition occurs, the shift of the Pb 4f peak is very limited [40, 41, 42]. Therefore, the 5 h UV treatment is considered to decomposes CsPbBr_3_ into CsBr and PbBr_2_. In Figure 4h, the Pb 4f binding energy remains unchanged, indicating that Pb^2+^ in the film has not been further oxidized. Therefore, UV treatment for 5 h causes CsPbBr_3_ to decompose into CsBr and PbBr_2_, which is a reversible process.

Considering that UV treatment is carried out in air, O_2_, H_2_O, and CO_2_ in the air can also participate in the reaction. To further explore the degradation mechanism, the UV exposure was extended to 24 and 72 h for additional XPS analysis. The spectra of Br 3d, Pb 4f, and Cs 3d reveal pronounced changes in chemical states with exposure times of 5, 24, and 72 h, as shown in Figure S15a–d. After 24 h, the binding energies of all three elements increase, and the Pb 4f and Cs 3d peaks become noticeably broader. After 72 h, these changes intensify, with both Pb 4f and Cs 3d exhibiting peak splitting. The Pb 4f peaks can be deconvoluted into three components: Pb─O bonds (137.97 eV), Pb─Br or Pb(OH)_2_ (138.60 eV), and PbCO_3_ (139.02 eV). The Cs 3d peaks are deconvoluted into two prominent peaks, including Cs─Br bonds (723.5 eV) and Cs_2_CO_3_ (725.0 eV). Notably, the peak area ratio between Br 3d_3/2_ and Br 3d_5/2_ decreased sharply from 1.13 to 0.03, indicating a significant reduction in Cs‐Br content. The emergence of PbO, PbCO_3_, and Cs_2_CO_3_ suggests that the final degradation products include these oxides and carbonates. The chemical degradation pathway of CsPbBr_3_ under UV exposure in ambient air is summarized as follows:

(4) $$\begin{array}{c} CsPbBr_{3}\;\to \;CsBr+PbBr_{2} \end{array}$$

(5) $$2\mathrm{CsBr}+H_{2}O+{\mathrm{CO}}_{2}\;\to \;{\mathrm{Cs}}_{2}{\mathrm{CO}}_{3}+2\mathrm{HBr}↑$$

(6) $$\begin{array}{c} PbBr_{2}\;\to \;Br_{2}↑+Pb^{2+}/Pb^{0+} \end{array}$$

(7) $$\begin{array}{c} 2Pb^{2+}/Pb^{0+}+H_{2}O+O_{2}\;\to \;PbO+Pb{\left(\mathrm{OH}\right)}_{2} \end{array}$$

(8) $$\begin{array}{c} 4HBr+O_{2}\;\to \;2H_{2}O+2Br_{2} \end{array}$$

This comprehensive chemical pathway of the light‐perovskite interaction is summarized in Figure 4j. In summary, 800 nm laser processing improves film morphology, crystallinity, and phase symmetry, thereby enhancing optoelectronic performance. In contrast, 365 nm UV exposure degrades the perovskite, resulting in deteriorated film performance due to chemical reactions over time. These reversible modifications underpin the optical “write–erase” mechanism.

This light‐induced reversible writing and erasing of CsPbBr_3_ thin films demonstrates strong potential for secure optoelectronic data encryption. Notably, a significant change in film thickness occurs only during the initial laser writing process. In subsequent “writing‐erasing” cycles, the thickness variation becomes negligible, while the optoelectronic response remains distinctly switchable. Visually, the laser‐written regions are indistinguishable from the UV‐erased ones, enabling invisible data encoding. In this system, the erased regions with low photocurrent responses represent binary “0,” whereas the laser‐written regions with high photocurrent responses correspond to binary “1.”

**FIGURE 5:**
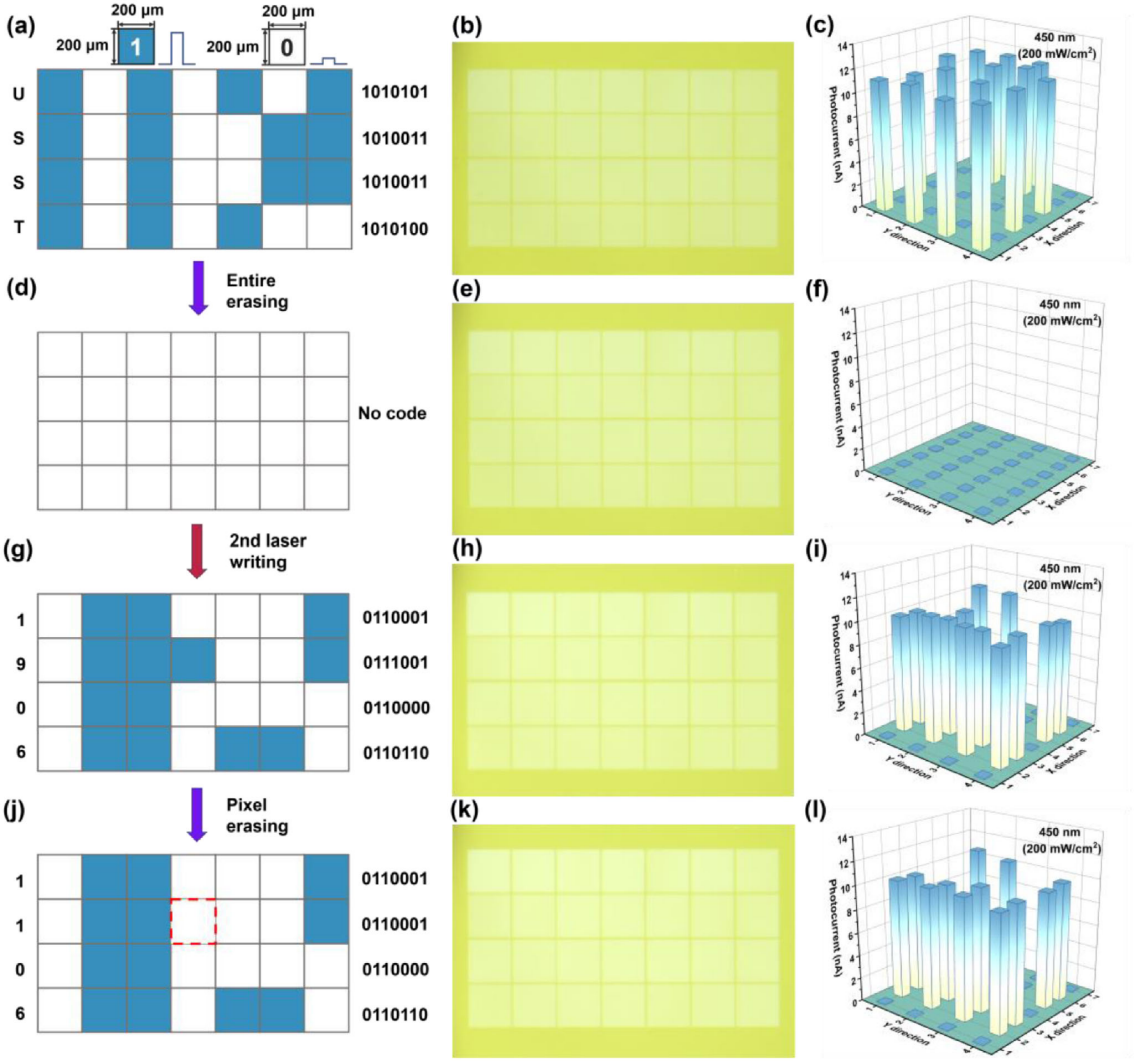
Light‐induced photocurrent change in CsPbBr_3_ thin film for encoding. (a,d,g,j) Schematic representation of the laser encoding (a), UV LED entire erasing (d), laser rewriting (g), and further UV laser partial erasing (j). The binary code is based on the high photocurrent signal from LIC indicating “1” and the low photocurrent signal from UV‐LIC indicating “0”. (b,e,h,k) Optical micrographs of the CsPbBr_3_ thin film after laser writing (b), UV LED erasing (e), laser rewriting (h), and UV laser partial erasing (k) demonstrate the invisibility of the encoded data. (c,f,i,l) Photocurrents of the device array displaying binary information on the film of the laser encoding (c), UV LED erasing (f), laser rewriting (g), and UV laser partial erasing (l).

Figure 5 illustrates the reversible optical encoding process on the perovskite thin film. A 7 × 4 pixel array was pre‐patterned using an 800 nm fs laser (300 mW, 10 µm/s), with each pixel 200 µm × 200 µm. The entire array was subsequently exposed to 365 nm UV light to erase all encoded information, resulting in a visually uniform array. As shown in Figure 5a, the text “USST” was first converted into a 7‐bit ASCII binary sequence and then patterned onto the array via laser writing (Figure 5b). Optoelectronic measurements across each pixel reconstructed the hidden message based on photocurrent intensity (Figure 5c). Upon UV illumination, the encoded information was completely erased (Figure 5d). The corresponding optical and photocurrent mappings are presented in Figure 5e–f.

To demonstrate rewriting capability, new data representing the number “1906” were converted to ASCII binary format and re‐encoded on the same array (Figure 5g). Figure 5h–i display the corresponding optical image and photocurrent mapping. Leveraging the local erasure capability of the UV beam, selective modification of a single pixel was achieved, converting “1906” to “1106” (Figure 5j). The associated microscope image and photocurrent diagram are shown in Figure 5k,l. Remarkably, no visible difference was observed in appearance after rewriting, yet the optoelectronic information was entirely updated.

This reversible optoelectronic encoding strategy offers several key advantages. The encrypted data remains invisible under optical inspection and is retrievable only through electrical readout, ensuring high‐level security. Furthermore, the precision of fs LDW allows dense data storage on miniaturized devices, achieving high information density. The capability to repeatedly erase and rewrite information significantly enhances device reusability and extends its operational lifetime, establishing a promising platform for secure, reconfigurable optoelectronic systems.

**FIGURE 6:**
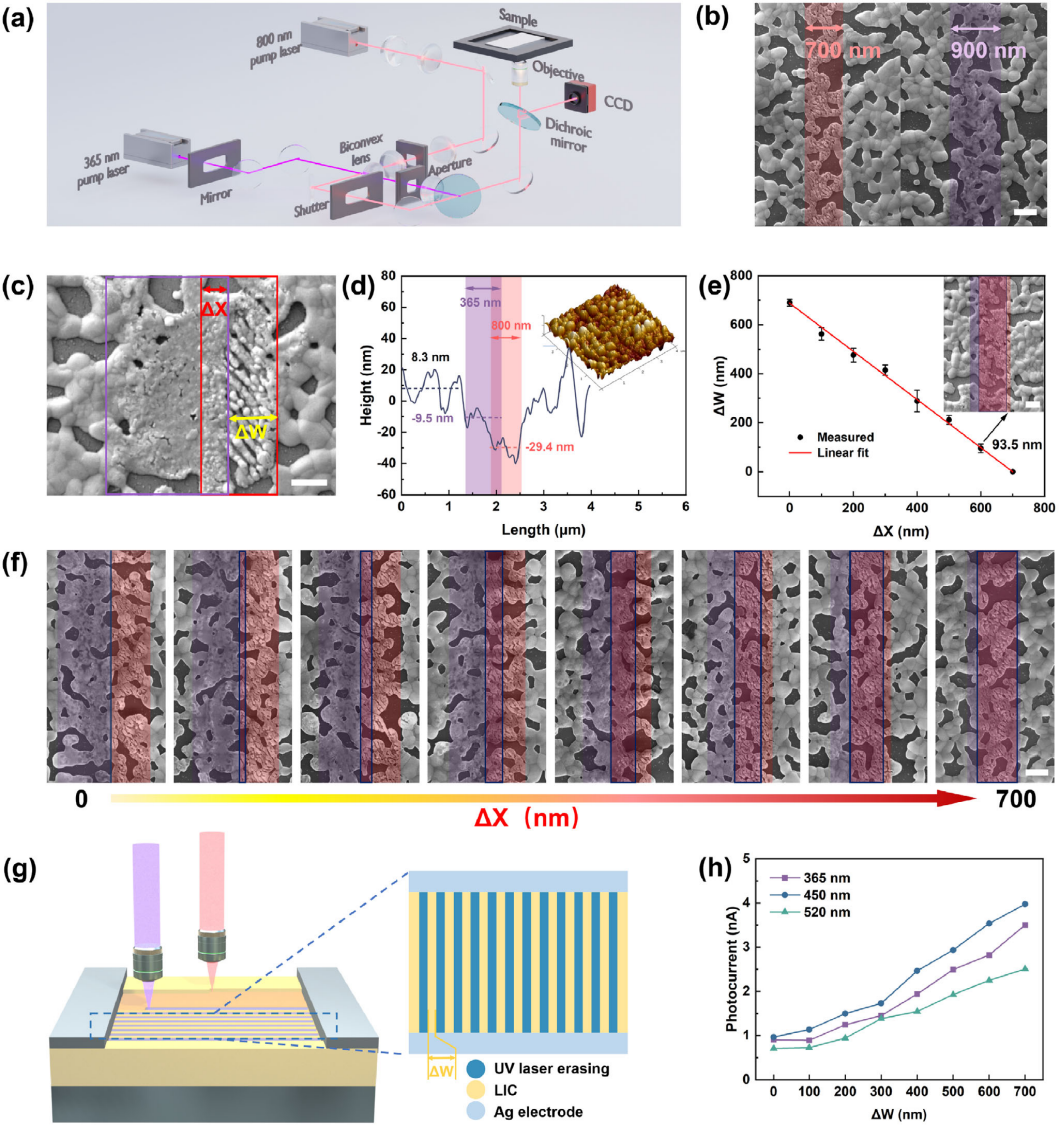
(a) Schematic diagram of the dual‐beam laser system with an 800 nm fs laser and a 365 nm UV laser process CsPbBr_3_ thin film. (b) SEM image of 800 nm fs laser writing CsPbBr_3_ thin film (left) and 365 nm UV laser erasing LIC (right). (c) SEM image of the sample, using the dual‐beam laser lithography with 200 nm overlap. (d) Height loss of the two laser beams lithography with 200 nm overlapping, and the inset shows the corresponding 3D AFM image. (e) The lithography linewidth of LIC with different overlaps between the two laser beams. The insect shows the SEM image of the sample, using two laser beams for lithography with a 600 nm overlap. (f) SEM image of the dual‐beam laser lithography with various overlapping. The scale bar of the SEM image: 400 nm. (g) Schematic diagram of the LIC linewidth adjustment through the dual‐beam laser system. (h) Photocurrent of the photodetector with different LIC linewidths. The power intensity of illumination: 365 nm (9 mW/cm^2^), 450 nm (200 mW/cm^2^), 520 nm (200 mW/cm^2^).

Figure 6a illustrates the dual‐beam laser lithography system consisting of an 800 nm fs writing laser and a 365 nm UV erasing laser, which was employed to fabricate LIC linewidths beyond the diffraction limit. Under optimized conditions, the 800 nm fs laser induced localized crystallization and phase transition of the CsPbBr_3_ film, producing a lithography linewidth of approximately 700 nm (Figure 6b). The introduction of the 365 nm UV laser enabled decomposition of the previously crystallized regions, thereby refining the linewidth of the LIC structures. As shown in Figure 6c, when the two laser beams overlapped by 200 nm, the linewidth decreased from 700 to 500 nm. The corresponding AFM image in Figure 6d confirms different etching depths produced by the two beams, with the 800 nm laser yielding a deeper ablation depth. By precisely adjusting the spatial overlap of the writing and erasing beams, the linewidth of LIC patterns can be continuously tuned from 700 nm down to 93.5 nm (Figure 6e). The colored SEM images corresponding to different overlappings are shown in Figure 6f. These results demonstrate that dual‐beam laser lithography enables controllable fabrication of perovskite LIC structures with tunable feature sizes, which can be used to modulate device performance. The linewidth (∆*W*) of LIC directly influences the photocurrent response of the CsPbBr_3_ photodetector. As depicted in Figure 6g, a 150 µm × 100 µm LIC region was first patterned using the 800 nm laser and subsequently erased by 365 nm laser irradiation. By varying the line spacing of the 365 nm laser, multiple LIC linewidths were achieved, resulting in multilevel regulation of photocurrent intensity (Figure 6h). The photocurrent of the devices increases proportionally with linewidth, where each linewidth corresponds to a distinct optoelectronic state.

## 3 Conclusion

In summary, we have developed a reversible optoelectronic modulation of CsPbBr_3_ perovskite thin films via 800 nm fs laser crystallization and 365 nm UV light‐induced decomposition. The 800 nm fs LDW induced localized recrystallization within the CsPbBr_3_ perovskite, leading to a phase transition from the orthorhombic to the cubic phase. This transition enhances crystallinity, reduces defects, and improves carrier transport, resulting in an over 100‐fold increase in photocurrent compared to the as‐prepared film. The fabricated LIC photodetector exhibits a broadband photoresponse from the UV to the visible spectrum, with the highest sensitivity to UV light at 365 nm. The laser crystallization of perovskite is triggered by the photothermal effect of the 800 nm fs laser. COMSOL simulations confirm that nonlinear multiphoton absorption during fs laser irradiation induces localized temperatures exceeding 800°C, initiating the crystallization process. To study the mechanism of this process, the 800 nm fs laser treatment has been compared with the thermal annealing process. Flexible LIC photodetectors were also fabricated on a temperature‐sensitive PET substrate and maintain excellent stability under repeated bending.

In contrast, 365 nm UV irradiation induced degradation of the CsPbBr_3_ perovskite into PbBr_2_ and CsBr, deteriorating its optoelectronic response. Time‐dependent UV exposure experiments reveal that CsPbBr_3_ undergoes reversible decomposition within 5 h of irradiation, while prolonged exposure beyond 24 h results in irreversible degradation. Thus, the decomposition behavior can be precisely tuned by controlling the UV exposure duration. Importantly, the photocurrent response of the CsPbBr_3_ films can be reversibly switched through alternating fs laser crystallization and UV erasing, with more than five repeatable cycles and 85% recovery efficiency. XRD, PL, Raman, and XPS analyses collectively confirm that this reversible modulation originates from structural phase transitions, defect passivation, and compositional changes.

Leveraging this reversible process, we further demonstrated an optoelectronic data encoding approach based on light‐induced crystallization and decomposition. Importantly, the encoding information is not visually observable and requires a specific optoelectronic readout for decryption, significantly enhancing data security. The arrayed perovskite structures demonstrate excellent endurance over multiple erasing and rewriting cycles. Compared to conventional data encryption strategies that rely on permanent patterning or electrical switching, our method offers a high‐resolution, reversible optical control platform. Moreover, laser writing provides precise spatial control and high data density, while UV erasing enables reusability, marking a significant step toward energy‐efficient, reconfigurable optoelectronic systems. In addition, a two‐beam laser system with an 800 nm fs laser and a 365 nm UV laser was employed to achieve feature patterns of perovskite beyond the diffraction limit barrier. The lithography linewidth of LIC can be modulated from 700 nm to subdiffraction 93.5 nm by partially overlapping the 800 nm writing laser and 365 nm erasing laser. The results pave the way toward super‐resolution lithography of perovskite, high‐density perovskite‐based devices, especially artificial intelligence devices with high neuron densities.

## 4 Methods

### 4.1 Materials and Chemicals

Lead (II) bromide (PbBr_2_, 99.999%) and cesium bromide (CsBr, 99.999%) were both purchased from Aladdin (Shanghai, China). Dimethyl sulfoxide (DMSO) was purchased from Sigma‐Aldrich Corp. 2 mmol CsBr and 2 mmol PbBr_2_ were dissolved in 5 mL DMSO, stirring at 70°C for 2 h. CsPbBr_3_ precursor solution was filtered by a polytetrafluoroethylene (PTFE) filter before use. A PTE with dimensions of 25 × 25 mm^2^ and a thickness of 0.125 mm is used as the substrate for a flexible photodetector.

### 4.2 Fabrication of CsPbBr_3_ Photodetector

We obtained the CsPbBr_3_ thin film through a one‐step spin‐coating method. The glass substrates were ultrasonically cleaned in acetone, ethanol, and deionized water for 15 min individually, dried with a nitrogen stream, and then placed in ultraviolet light for 15 min. Next, the perovskite precursor was spin‐coated on the glass substrate at 2000 rpm for 60 s to form the CsPbBr_3_ thin film. Ag electrodes with a thickness of 60 nm were grown on the CsPbBr_3_ thin film by thermal deposition, forming a metal‐semiconductor‐metal (MSM) structure. An 800 nm fs laser (80 MHz) was applied to scan the fabricated CsPbBr_3_ thin film between the Ag electrodes for photodetector applications. The corresponding power density for an 800 nm femtosecond laser with a power of 300 mW is 3.82 × 10^7^ W/cm^2^, and corresponding energy density is 477.5 mJ/cm^2^. The processed region measures 150 µm × 50 µm. The CsPbBr_3_ flexible photodetectors are fabricated using the same process. A 365 nm UV LED from Thorlabs (M365L3) with a power density of 200 mW/cm^2^ was applied. A 365 nm UV laser with a continuous wave was used, with a maximum laser power of 25 mW. The corresponding power density for a 365 nm UV laser with a power of 10 mW is 1.273 × 10^6^ W/cm^2^.

### 4.3 Measurement

The devices were measured without being encapsulated. All the devices’ performance measurements were conducted at ambient conditions. The current–voltage (*I*–*V*) and current–time (*I*–*T*) characteristics were measured on a probe station, and the data were recorded by a semiconductor parameter analyzer (Keithley 4200A‐SCS).

### 4.4 Characterization

Scanning electron microscope (SEM) images were obtained using the ZEISS Crossbeam 550 Gemini 2 instrument. The atomic force microscope (AFM) images were obtained using Bruker Dimension ICON. The crystal structure by x‐ray diffraction (XRD) was characterized with a Rigaku SmartLab SE. The x‐ray photoelectron spectroscope (XPS, Thermo K‐Alpha) was used to determine the chemical composition and bonding configurations of cesium, lead, and bromine atoms. Photoluminescence (PL) spectroscopy was carried out with an Andor Shamrock 500i imaging spectrometer, equipped with a Leica DFC295 Digital Color Camera and excited by a laser at a wavelength of 405 nm. UV–visible absorption spectroscopy was performed using an Andor Shamrock 500i spectrometer equipped with a CCD detector (DU970P‐BVF). Time resolved photoluminescence (TRPL) was measured by Leica Stellaris 8 STED confocal scanning microscope (Leica microsystems, Germany).

## Conflicts of Interest

The authors declare no conflicts of interest.

## Supporting information




**Supporting File**: adma73939‐sup‐0001‐SuppMat.docx.

## Data Availability

The data that support the findings of this study are available from the corresponding author upon reasonable request.
