# Supplementary material for: Sub‐Diffraction Nanolithography of Halide Perovskite via Reversible All‐Optical Crystallization–Decomposition
Source: Adv Mater. 2026 Jul 4;38(44):e73939. doi: 10.1002/adma.73939 (PMC13449109; doi:10.1002/adma.73939)
Supplement: Supplementary file 1 [file ADMA-38-e73939-s001.docx]

## **Supplementary Information**

**Figure S1:** The optical path of the laser-processing system.

**Figure S2** The stability of the photodetector under long-term illumination of 365 nm light at a low power density (9 mW/cm^2^) for 2 hours.

**Figure S3** Photocurrents of LIC photodetectors fabricated with different laser processing parameters under the irradiation of (a) 450 nm, (b) 520 nm, (c) 638 nm.

**Figure S4** The photocurrent of the CsPbBr_3_ photodetectors prepared with lower laser parameters.

**Figure S5** I-T curves of UC-based photodetector irradiated by (a) 365 nm, (b) 450 nm, (c) 520 nm, and (d) 638 nm light with various intensities.

**Figure S6** I-T curves of LIC photodetector irradiated by (a) 365 nm, (b) 450 nm, (c) 520 nm, and (d) 638 nm light with various intensities.

**Figure S5a-d** presents the I-T characteristics of the UC-based photodetector under illumination of different wavelengths. The device exhibits stable photocurrent pulse responses, with photocurrent amplitude increasing as the light power density rises. **Figure S6a-d** shows the corresponding results for the LIC-based photodetector. After laser processing, the LIC device exhibits a significant improvement in photocurrent. It displays an approximately 100-fold enhancement in photocurrent response at 365 nm, while maintaining excellent stability across a range of wavelengths.

**Figure S7** The responsivity (R) and external quantum efficiency (EQE) of the LIC-based photodetector for (a) 365 nm, (b) 450 nm, and (c) 520 nm light irradiation. The specific detectivity (D*) of the LIC-based photodetector under (d) 365 nm, (e) 450 nm, and (f) 520 nm light irradiation.

**Table S1** Summary of the device performances of the CsPbBr_3_-Based photodetectors.

| **Device structure** | **Fabrication** | **Wavelength (nm)** | **R (A/W)** | **EQE (%)** | **D* (Jones)** | **Ref.** |
| --- | --- | --- | --- | --- | --- | --- |
| Ag/LIC/Ag | Laser-induced | 365 | 0.0124 | 4.22 | 1.86 × 10^9^ | This work |
| ITO/ZnO:CsPbBr_3_/Ag | Doping | 532 | 0.000141 | / | 4.43 × 10^7^ | ^1^ |
| ITO/CsPbBr_3_/PMMA/Ag | Evaporation | 532 | 0.11 | / | 4.4 × 10^10^ | ^2^ |
| FTO/c-TiO_2_/CsPbBr_3_ nanocrystals/Au | Solution | 360 | 0.00221 | / | 2.89 × 10^9^ | ^3^ |
| Au/CsPbBr_3_:CsPb_2_Br_5_/Au | Evaporation | 405 | 0.206 | / | 4.07 × 10^9^ | ^4^ |
| Au/CsPbBr_3_ microrod single crystals/Au | Solution | 405 | 0.025 | 7.68 | 3.67 × 10^12^ | ^5^ |
| FTO/ZnO_2_:CsPbBr_3_/Carbon | Doping | 473 | 0.1 | / | 4.2 × 10^12^ | ^6^ |
| FTO/NiOx/CsPbBr_3_/TiO2/Au | Solution | 405 | 0.05 | / | 1.59 × 10^13^ | ^7^ |
| ITO/SnO_2_/CsPbBr_3_/MoO_3_/Au | Evaporation | 473 | 0.09 | / | 7.44 × 10^11^ | ^8^ |
| FTO/TiO_2_/WS_2_:CsPbBr_3_/Ag | Doping | 450 | 0.003 | / | 3.7 × 10^11^ | ^9^ |
| ITO/SnO_2_/CsPbBr_3_/P3HT/Carbon/Cu | Solution | 525 | 0.34 | 83 | 1.31 ×10^12^ | ^10^ |

Responsivity (R) represents the ability of the photodetector to convert light into electrical signal, external quantum efficiency (EQE) is the ratio of the number of photogenerated electrons to the number of incident photons, and detectivity (D*) can represent the ability of the device to detect weak light signals. The above-mentioned three key parameters could be calculated using the following equations:

$$\begin{aligned} R=\frac{I_{l}-I_{d}}{PS}\#(1) \end{aligned}$$

$$\begin{aligned} EQE=\frac{Rhc}{e\lambda}\#(2) \end{aligned}$$

$\begin{aligned} D^{*}=\frac{R\sqrt{S}}{\sqrt{2eI_{d}}}\#(3) \end{aligned}$Where $I_{l}$ is the photocurrent, $I_{d}$ is the dark current, $P$ is the illumination power density, *h* is Planck's constant, *c* is the velocity of light, *λ* is the wavelength of the incident light, and *e* is the elementary charge.

**Figure S8** (a) Absorbance spectra, (b) PL spectra, and (c) XRD spectra of the laser annealed sample and the thermal annealed samples at 70 °C, 100 °C, and 130 °C.

The laser-annealed and thermally annealed samples were characterized to compare their optical and structural properties. **Figure S8a** shows the absorption spectra of the samples. All samples share an absorption edge around 540 nm with nearly identical peak positions, and the laser-annealed sample exhibits a stronger absorption peak, indicating that laser processing does not alter the intrinsic optical absorption characteristics of the perovskite. **Figure S8b** presents the steady-state PL spectra of the samples. The PL intensity decreases after both thermal and laser annealing, with the LIC sample showing the weakest emission. This reduction is attributed to the formation of larger grains after laser annealing, which suppresses excitonic radiative recombination. A noticeable blue shift of the PL peak in CsPbBr₃ after annealing suggests an increased bandgap, likely due to the reduction of shallow-level defects acting as non-radiative recombination centers. **Figure S8c** displays the XRD patterns of the samples, which show characteristic perovskite diffraction peaks confirming the formation of the perovskite phase. Notably, the laser-annealed sample exhibits sharper and more intense diffraction peaks, indicating higher crystallinity.

**Figure S9** Characterization comparison of CsPbBr_3_ films annealed at different temperatures (160-240 °C) (a) Photocurrent responses of the three samples under 450 nm illumination at 200 mW/cm^2^. (b) The XRD spectra of the three samples. (c) The XPS survey spectra of the three samples. High-resolution XPS spectra of the three samples: (d) Br 3d, (e) Pb 4f, (f) Cs 3d.

To verify the effect of high-temperature annealing on CsPbBr_3_ thin films, we annealed the films at 160 °C, 200 °C, and 240 °C for 15 min, respectively, and compared the film performance under different annealing temperatures. **Figure S9a** shows the photocurrent responses of the three films under 450 nm illumination at a power density of 200 mW/cm^2^. The results show that the photocurrent response gradually decreases with increasing annealing temperature, indicating that high-temperature annealing degrades the optoelectronic performance of the perovskite films. **Figure S9b** presents the XRD patterns of CsPbBr_3_ films annealed at different temperatures. Although characteristic diffraction peaks of CsPbBr_3_ are observed in all samples, the peak intensity significantly decreases as the annealing temperature rises, indicating reduced crystallinity. XPS analysis (**Figure S9c-f**) also reveals a similar trend of peak intensity attenuation, suggesting that high-temperature annealing induces mass loss in the CsPbBr_3_ films, thereby leading to performance degradation.

**Figure S10** Comparison of photocurrent for two annealing methods: laser annealing and thermal annealing. The photocurrent of the laser annealed sample and the thermal annealed samples at 70°C, 100°C, 130°C, irradiated by (a) 365 nm, (b) 450 nm, and (c) 520 nm light.

**Figure S10a-c** shows the photocurrents generated by the laser-annealed and thermally annealed samples under illumination at 365 nm, 450 nm, and 520 nm, respectively. Under all these wavelengths, the laser-annealed samples exhibit higher photocurrents than the thermally annealed ones. Owing to the phase transitions in the perovskite crystal structure induced by different annealing temperatures, the thermally annealed sample treated at 100 °C shows the highest photocurrent. In comparison, the sample annealed at 70 °C displays the lowest.

**Figure S11** (a) The definition of bending degrees (θ). (b) The photograph of the LIC-based flexible photodetector with various bending degrees. (c) The I-V curve of the LIC-based flexible photodetector irradiated by various wavelengths. (d) The I-T curve of the LIC-based flexible photodetector with various bending degrees. (Illumination: 450 nm, 200 mW/cm^2^). (e) The I-V curve of the LIC-based flexible photodetector after 0-300 bending cycles. (f) R and D* of LIC-based flexible photodetector after 0-300 bending cycles. (g) The stability of the LIC-based flexible photodetector**.**

A CsPbBr₃ film was fabricated on a 0.125 mm-thick PET substrate, followed by the deposition of a 60 nm Ag electrode. The flexible LIC-based photodetector was then fabricated with 800 nm fs laser processing. **Figure S11a shows the definition of bending degrees(θ). Figure S11b** shows photographs of the LIC-based flexible photodetector under different bending conditions. **Figure S11c** shows the I-V curves of the LIC-based flexible photodetector irradiated by various wavelengths. The photocurrent response characteristics are consistent with the previous results. The I-T characteristics were measured at multiple bending curvatures, as shown in **Figure S11d.** The photocurrent of the device increases with the degree of bending. Subsequently, a stability test was conducted on the photodetector. After 300 cycles of bending, the photodetector still maintained excellent performance, as shown in **Figure S11e-f.** After the photodetector was stored in the argon chamber for 35 days, its photocurrent remained above 95% of the original value, as shown in **Figure S11g**.

**Figure S12** The absorption coefficient of UC, LIC and UV-LIC films.

**Figure S13** (a) The SCLC measurements and (b) fitted TRPL curves of the UC, LIC and UV-LIC.

Space-Charge-Limited Current (SCLC) was used to evaluate the defects of the UC, LIC and UV-LIC as shown in **Figure S13a**. The trap-filled limit voltages (VTFL) are 0.28 V, 0.21 V and 0.31 V for the UC, LIC and UV-LIC, respectively. Based on VTFL values, the defect state density (n_trap_) of the perovskite films can be expressed by:

$$\begin{aligned} n_{trap}=\frac{2\varepsilon_{0}\varepsilon_{r}V_{TFL}}{qL^{2}}\#(4) \end{aligned}$$

where $\varepsilon_{0}$is the vacuum dielectric constant, $\varepsilon_{r}$ is the relative dielectric constant of inorganic chalcogenides, $q$ is the meta-charge, and $L$ is the film thickness. The defect density of LIC film is 9.57 × 10^15^ cm^−3^ which are less than the UC film (1.28 × 10^16^ cm^−3^) and UV-LIC (1.41 × 10^16^ cm^−3^).

**Figure S14** Survey XPS spectra of the UC, LIC, and UV-LIC.

**Figure S15** (a) Survey XPS spectra of the UV-LIC with different UV-treatment times: 5 hours, 24 hours, and 72 hours. High-resolution XPS spectra corresponding to (b) Br 3d, (c) Pb 4f, and (d) Cs 3d for different UV-treatment durations.

**Figure S15a** presents the survey XPS spectra of the CsPbBr₃ films after UV treatment for 5, 24, and 72 hours, while **Figure S15b-d** shows the corresponding high-resolution spectra of Br 3d, Pb 4f, and Cs 3d, respectively. Evident changes in the chemical states of Br, Pb, and Cs are observed with increasing UV exposure duration. After 24 hours of UV irradiation, the Br 3d, Pb 4f, and Cs 3d peaks shift toward higher binding energies, accompanied by a noticeable broadening of the Pb 4f and Cs 3d peaks. Upon 72 hours of UV treatment, these effects become more pronounced, and distinct peak splitting appears in the Pb 4f and Cs 3d spectra. The Pb 4f region can be deconvoluted into three components: the peak at 137.97 eV corresponds to Pb–O bonds, the one at 138.60 eV is attributed to Pb–Br bonds or Pb(OH)₂, and the peak at 139.02 eV arises from PbCO₃. Similarly, the Cs 3d spectrum can be fitted with two peaks: one at 723.5 eV corresponding to Cs–Br bonds and another at 725 eV associated with Cs₂CO₃. Notably, the area ratio of the Br 3d₃/₂ to Br 3d₅/₂ peaks decreases significantly, indicating a substantial reduction in Cs–Br content due to Br loss in the form of Br₂ and HBr gases. Combined with the observed peak splitting in the Pb 4f and Cs 3d spectra, these results confirm the formation of degradation products such as PbO, PbCO₃, and Cs₂CO₃ after prolonged UV exposure.

**Figure S16** The AFM images of (a) UC, (b) LIC, and (c) UV-LIC (scale bar: 200 nm). The corresponding 3D AFM images of (d) UC, (e) LIC, and (f) UV-LIC.

**Figure S16** presents the AFM images, which show the surface morphology and roughness of the UC, LIC, and UV-LIC samples. The LIC film exhibits a surface roughness of 1.19 nm, lower than that of the UC sample (2.00 nm), whereas the UV-LIC film shows a significantly higher roughness of 17.4 nm. These results indicate that laser processing smooths the surface of perovskite film, while UV irradiation substantially increases its surface roughness. The three-dimensional AFM images further support these observations. A smoother perovskite surface facilitates carrier transport and reduces non-radiative recombination sites, thereby suppressing spontaneous electron-hole recombination and enhancing the overall performance of the perovskite film.

**Figure S17** (a) The effect of annealing temperature on recovery outcome. (b) Comparison of photocurrent for two recovery methods: Thermal anneal and Laser recovery.

By comparing the photocurrent of the photodetector, the recovery effect of various thermal annealing temperatures on the performance of the photodetector is evaluated. **Figure S17a** shows the photocurrent of the UV-LIC photodetector after annealing at different temperatures. The results indicate that the optimal temperature for thermal annealing recovery is 100°C. Subsequently, the effects of 100°C annealing, laser recovery, and post-anneal laser were compared, as shown in **Figure S17b.** It can be observed that the photocurrent of the UV-LIC photodetector can be recovered using various methods, including thermal annealing, laser annealing, and a combination of laser and thermal annealing. After post-anneal laser recovery (thermal annealing following the laser process), the sample exhibits the highest recovery, followed by laser recovery and thermal annealing.

**Figure S18** (a-b) The calculated hologram (a) and the designed image (b). (c) The microscopic image of the laser patterned on CsPbBr_3_ thin film, and (d) the image of its reconstructed hologram.

The calculated hologram and designed image were shown in **Figure S18a-b**. The laser-written perovskite hologram was fabricated using an 800 nm laser with a power of 200 mW, and reconstructed in the optical system, displaying the word “IPC” in **Figure S18c-d**.

**Figure S19** The SEM images of UV-LIC processed by 365 nm laser with various intensities.

To evaluate the feasibility of UV laser “erasing”, the LIC samples were locally irradiated with 365 nm lasers at different powers. **Figure S19** shows SEM images of the LIC films after processing with varying laser powers. Under 365 nm irradiation, the CsPbBr₃ perovskite in the film decomposes, forming numerous nanoscale voids, while Pb⁰/Pb²⁺ species precipitate as small particles distributed on the film surface. As the laser power increases, the degree of perovskite decomposition becomes more pronounced.

# Modeling of the Laser-induced treatment process

**Figure S20** Two-dimensional model of perovskite photodetector for simulation.

## **Geometry and materials**

The model geometry consists of two layers: glass and perovskite. The thicknesses of these layers were measured with a profilometer. The absorption coefficients of the aforementioned materials were measured directly using a UV-vis spectrometer. Other properties of these materials were imported from the COMSOL library and manually from relevant studies (Phillips et al., 2016; Saxena and Gorji, 2019), as described in Table S2. The initial temperature of the model is set at 25 °C, which corresponds to room temperature.

## **Physic**

To investigate the underlying mechanisms of the laser heating process, a Radiative Beam in Absorbing Media was performed using a commercially available finite element software package (COMSOL Multiphysics 6.0). **Figure S15** shows the two-dimensional (2D) models used in this study. When the laser beam with a Gaussian distribution hits the surface of the perovskite layer, a portion of the laser energy is absorbed and converted into instantaneous local heating. The differential equation describing the thermal effects is the heat-transfer equation:

$$\rho C_{p}\frac{\partial T}{\partial t}+\rho C_{p}\mathbf{u}\cdot\nabla T+\nabla\cdot\mathbf{q}=Q$$

(4)

$$\mathbf{q}=-k\nabla T$$

(5)

When light is incident upon a semitransparent material, some of the energy will be absorbed by the material itself. It is assumed that the laser light is a single wavelength and experiences minimal refraction, reflection, or scattering within the material itself; therefore, it is appropriate to model the light intensity using the Beer-Lambert law. This law can be written in differential form for the light intensity as:

$$\frac{\partial I}{\partial y}=\alpha(T)I$$

(6)

Where $\alpha$ is the absorption coefficient of the materials, the incident pulsed laser beam is distributed with a Gaussian shape in time and space according to the following equation:

$$I=\frac{P_{0}}{2\pi\sigma^{2}}\exp(-\frac{\left( x\left( t \right)-x_{0} \right)^{2}}{2\sigma^{2}})$$

(7)

The laser beam is moved along the X-axis at a speed of 10 μm/s, with a repetition rate of 5 kHz and a power of 300-500 mW. The spot size of the laser beam is 500 nm**.** These values were calculated according to the following equation:

$$d\left( z \right)={2w}_{0}\sqrt{1+\frac{z}{z_{R}}}$$

Where $z_{R}=\frac{\pi w_{0}^{2}}{\lambda}$ is the Rayleigh length, w_0_ is the focal radius (500 nm), and λ is the wavelength of the laser beam.

The heat source term can be written as follows by considering the material reflectivity, the absorption coefficient of the material, and the laser intensity:

$$Q=\left( 1-R\left( T \right) \right)\alpha(T)I$$

(8)

All terms of equations (1), (2), (3), (4), and (5) are defined in Table S3.

Table S2. Simulation parameters

|  | Perovskite | Glass |
| --- | --- | --- |
| Thickness (nm) | 200 | 4000 |
| $C_{p}$ (J/kg.K) | 224 | 730 |
| $\rho$ (kg/m^3^) | 3890 | 2210 |
| $k$ (W/m.K) | 0.5 | 1.56 |
| α | 1670 | 0 |

Table S3. Nomenclature

| $C_{p}$ | Material heat capacity at constant pressure ($j/(kg\cdot K\mathbf{)}$) |
| --- | --- |
| $k_{0}$ | Free-space wavenumber ($m^{-1}$) |
| q | Heat flux vector (W/m^2^) |
| Q | Heat source ($W/m^{3})$ |
| u | Velocity vector of translation motion (m/s) |
| $T$ | Temperature (°K) |
| $k$ | Thermal conductivity ($\frac{W}{m\cdot K}$) |
| $\rho$ | Material density ($\frac{\mathrm{kg}}{m^{3}}$) |
| $\sigma$ | Standard deviation of the Gaussian distribution |
| α | Absorption coefficient (cm^-1^) |

1.	Nagal, V.*, et al.*, Insight into Hot Carrier Kinetics of CsPbBr_3_/ZnO Heterostructures for Photodetector Application, ***ACS Applied Optical Materials*** 1, 779-787 (2023).

2.	Liu, X.*, et al.*, Ultrafast, self-powered and charge-transport-layer-free photodetectors based on high-quality evaporated CsPbBr_3_ perovskites for applications in optical communication, ***Journal of Materials Chemistry C*** 8, 3337-3350 (2020).

3.	Suhail, A., Saini, A., Beniwal, S., Bag, M., Tunable Optoelectronic Properties of CsPbBr_3_ Perovskite Nanocrystals for Photodetectors Applications, ***The Journal of Physical Chemistry C*** 127, 17298-17306 (2023).

4.	Sun, Z.*, et al.*, Nanometer-Thick CsPbBr_3_ Films with Embedded CsPb_2_Br_5_ Nanowires for Photodetector Applications, ***ACS Applied Nano Materials*** 7, 11785-11793 (2024).

5.	Cheng, X.*, et al.*, Nucleation-controlled growth of superior long oriented CsPbBr_3_ microrod single crystals for high detectivity photodetectors, ***Journal of Materials Chemistry C*** 7, 14188-14197 (2019).

6.	Pan, X.*, et al.*, Single-Layer ZnO Hollow Hemispheres Enable High-Performance Self-Powered Perovskite Photodetector for Optical Communication, ***Nano-Micro Letters*** 13, 70 (2021).

7.	Ji, Z.*, et al.*, All‐Inorganic Perovskite Photodetectors with Ultrabroad Linear Dynamic Range for Weak‐Light Imaging Applications, ***Advanced Optical Materials*** 8, 2001436 (2020).

8.	Wang, R.*, et al.*, Self-Powered CsPbBr_3_ Perovskite Nanonet Photodetector with a Hollow Vertical Structure, ***Journal Physical Chemistry Letters*** 12, 7519-7525 (2021).

9.	Nayak, S. K.*, et al.*, Critical Role of Carrier Cooling Mechanism in WS_2_/CsPbBr_3_ Hybrid Nanocomposites for Enhanced Photodetector Performances, ***Small*** 21, e2410099 (2025).

10.	Deng, Y.*, et al.*, Enhanced Bandwidth of Blue‐Green‐Light CsPbBr_3_ Perovskite Photodetectors toward Underwater Wireless Optical Communication, ***Laser & Photonics Reviews*** 19, e01332 (2025).
